# Nanosecond electric pulses differentially affect inward and outward currents in patch clamped adrenal chromaffin cells

**DOI:** 10.1371/journal.pone.0181002

**Published:** 2017-07-10

**Authors:** Lisha Yang, Gale L. Craviso, P. Thomas Vernier, Indira Chatterjee, Normand Leblanc

**Affiliations:** 1 Department of Pharmacology, University of Nevada, Reno School of Medicine, Reno, NV, United States of America; 2 Frank Reidy Research Center for Bioelectrics, Old Dominion University, Norfolk, VA, United States of America; 3 Department of Electrical and Biomedical Engineering, College of Engineering, University of Nevada, Reno, NV, United States of America; 4 Center for Cardiovascular Research, University of Nevada, Reno School of Medicine, Reno, NV, United States of America; Consiglio Nazionale delle Ricerche, ITALY

## Abstract

This study examined the effect of 5 ns electric pulses on macroscopic ionic currents in whole-cell voltage-clamped adrenal chromaffin cells. Current-voltage (I-V) relationships first established that the early peak inward current was primarily composed of a fast voltage-dependent Na^+^ current (I_Na_), whereas the late outward current was composed of at least three ionic currents: a voltage-gated Ca^2+^ current (I_Ca_), a Ca^2+^-activated K^+^ current (I_K(Ca)_), and a sustained voltage-dependent delayed rectifier K^+^ current (I_KV_). A constant-voltage step protocol was next used to monitor peak inward and late outward currents before and after cell exposure to a 5 ns pulse. A single pulse applied at an electric (E)-field amplitude of 5 MV/m resulted in an instantaneous decrease of ~4% in peak I_Na_ that then declined exponentially to a level that was ~85% of the initial level after 10 min. Increasing the E-field amplitude to 8 or 10 MV/m caused a twofold greater inhibitory effect on peak I_Na_. The decrease in I_Na_ was not due to a change in either the steady-state inactivation or activation of the Na^+^ channel but instead was associated with a decrease in maximal Na^+^ conductance. Late outward current was not affected by a pulse applied at 5 MV/m. However, for a pulse applied at the higher E-field amplitudes of 8 and 10 MV/m, late outward current in some cells underwent a progressive ~22% decline over the course of the first 20 s following pulse exposure, with no further decline. The effect was most likely concentrated on I_Ca_ and I_K(Ca)_ as I_KV_ was not affected. The results of this study indicate that in whole-cell patch clamped adrenal chromaffin cells, a 5 ns pulse differentially inhibits specific voltage-gated ionic currents in a manner that can be manipulated by tuning E-field amplitude.

## Introduction

Exposing biological cells to nanosecond-duration, high-intensity (>1 MV/m) electric pulses (NEPs) causes effects on the plasma membrane conductance properties of cells by forming nanometer-diameter pores (nanopores) in the lipid bilayer [1–3]. Ion-conducting electropores formed in response to NEPs are membrane-permeabilizing structures that exhibit complex ion channel-like conductance that can last for minutes [3–6]. In addition, the very short duration of the pulses allows the electric field to penetrate the plasma membrane and cause intracellular effects, such as the release of calcium from internal stores [7–9] that can trigger various cell responses.

Depending on the cell type, NEP-evoked nanopores can cause cell swelling from osmotic imbalance [10–12]. Cell swelling has been observed for NEPs ranging in duration from 600 ns to as short as 5 ns [13–17]. In excitable adrenal chromaffin cells exposed to 5 ns pulses, cell swelling does not occur [18]. Instead, the main effect of plasma membrane nanoporation is that of a cell stimulus to evoke catecholamine release. When these cells are exposed to a single 5 ns, 5 MV/m pulse, voltage-gated Ca^2+^ channels (VGCCs) are activated, resulting in Ca^2+^ influx that triggers catecholamine release by exocytosis [19–21]. VGCC activation has been attributed to plasma membrane depolarization that is mediated by Na^+^ influx via ion-conducting nanopores [20]. Whole-cell patch clamp recordings support this mechanism by showing that a single 5 ns pulse induces an instantaneous inward current that is carried, at least in part, by Na^+^, and which does not involve voltage-gated Na^+^ channels [22]. Thus, Na^+^ influx via plasma membrane nanopores could serve as an alternative depolarizing mechanism typically performed physiologically by activation of cation-permeable nicotinic receptors and subsequent stimulation of voltage-gated Na^+^ channels [23].

Although a 5 ns pulse can alter chromaffin cell excitability by allowing Na^+^ to cross the plasma membrane via nanopores, overall cell excitability could be further affected if the pulse also exerted effects on voltage-gated ion channels. In this regard, Pakhomov et al. [24,25] found that along with plasma membrane permeabilization, longer duration pulses (300 and 600 ns) exerted a prolonged inhibitory effect on voltage-gated Na^+^ and Ca^2+^ channels in GH_3_ cells, NG108 cells and adrenal chromaffin cells. Whether a pulse of only 5 ns in duration could also alter ionic channels is still unexplored and was the purpose of this study. To this end, we carried out whole-cell patch clamp experiments in chromaffin cells to determine the effect of a single 5 ns pulse on macroscopic ion currents, which in these cells comprise a mixture of Na^+^, Ca^2+^ and K^+^ currents [26,27]. Our experimental approach utilized conditions designed to simulate near physiological ion gradients and a whole-cell recording system in which effects of a NEP on macroscopic inward and outward currents were monitored 0.5 s after the pulse was applied to the cells.

## Materials and methods

### Chromaffin cell culturing and preparation

Adrenal chromaffin cells were isolated by collagenase digestion of the medulla of fresh bovine adrenal glands obtained from a local abattoir (Wolf Pack Meats, University of Nevada, Reno) and maintained in suspension culture in Ham’s F-12 medium supplemented with 10% bovine calf serum, 100 U/ml penicillin, 100 μg/ml streptomycin, 0.25 μg/ml fungizone, and 6 μg/ml cytosine arabinoside at 36.5°C under a humidified atmosphere of 5% CO_2_ as previously described [18–22]. Cells were used up until 14 days in culture. For experiments, large cell clusters were dissociated into single isolated cells with the protease dispase [28] and attached to fibronectin-coated glass coverslips [22]. Once attached, cells retained their spherical morphology and were used either the same day or for a period not exceeding two days.

### Patch clamp electrophysiology

Coverslips containing the attached cells were placed inside a perfusion chamber that was mounted on the stage of an inverted Nikon Eclipse TS100 microscope. The chamber was continuously perfused at a rate of 0.5 ml/min with a balanced salt solution (BSS) consisting of 145 mM NaCl, 5 mM KCl, 2 mM CaCl_2_, 1.2 mM Na_2_HPO_4_, 1.3 mM MgCl_2_, 10 mM glucose and 15 mM Hepes, pH 7.4 at room temperature. Whole-cell currents were monitored in voltage-clamp mode using an Axopatch 200B amplifier and Digidata 1322A data acquisition system (Axon Instruments, Sunnyvale, CA), and pClamp software (version 8.2, Molecular Devices, Sunnyvale, CA) at a sampling rate of 20 kHz and low-pass filtering at 1 kHz. The series resistance (Rs), which was compensated to 90%, varied between 6 and 28.3 MΩ (average 11.7 ± 1.1 MΩ, n = 21) and the seal resistance (Rm) varied between 1.3 and 3.2 GΩ (average 2.2 ± 0.1 GΩ, n = 21). Cell capacitance (Cm) ranged between 4.2 and 13.7 pF (average 8.8 ± 0.4 pF, n = 21). Micropipettes having a tip size of 0.8–1.2 μm were fabricated from borosilicate glass (#BF150-110-7.5, Sutter Instruments, Novato, CA) using a P-97 pipette puller (Sutter Instruments, Novato, CA) and a MF-830 microforge (Narishige, Tokyo, Japan). The patch pipette internal solution contained 10 mM NaCl, 30 mM KCl, 110 mM K-gluconate, 1 mM MgCl_2_, 10 mM EGTA, 3 mM Mg.ATP, and 10 mM Hepes, pH 7.2 (adjusted with KOH) at room temperature. The cell being recorded was viewed with a 40X objective and bright field images of the cells were captured with a CoolSnap HQ DIFF CCD camera (Photometrics, Tucson, AZ) and SimplePCI software (version 6.6.0.0, Hamamatsu Corporation, Hamamatsu City, Japan) at the start and end of experiments. For recordings obtained in the absence of extracellular Na^+^, Na^+^ in the external bath solution was replaced with an equimolar concentration of N-methyl-D-glucamine (NMDG^+^). For experiments conducted in the absence of both extracellular Ca^2+^ and Na^+^, the external NMDG^+^-containing bath solution lacked Ca^2+^. A 3 M KCl-agar salt bridge was used to minimize changes in liquid junction potentials when changing the external solution from BSS to NMDG^+^ or to Ca^2+^-free NMDG^+^ solutions [29,30]. The salt bridge was gelled in 4% (w/v) agar and enclosed in a U-shaped microhematocrit capillary tube. In all experiments, the holding potential (HP) was set to –70 mV.

Current-voltage (I-V) relationships for inward and outward currents were generated using a voltage step protocol consisting of 50 ms steps ranging from –70 mV to +80 mV applied in 10 mV increments every 2 s. NEP effects on peak inward and late outward currents were monitored using a constant-voltage step protocol in which voltage steps to +10 mV or to +80 mV were applied every 3 seconds from a HP of –70 mV. Steady-state activation curves for Na^+^ currents were constructed by measuring the peak Na^+^ conductance (G_Na_) calculated from the equation:

$$G_{Na}=\frac{I_{Na}}{V-E_{rev}},$$

where I_Na_ is the peak Na^+^ current during the test depolarization (V), and E_rev_ is the reversal potential of the inward current. Data were normalized to maximum peak conductance (G_max_) and fitted to a Boltzmann function:

$$\frac{G_{Na}}{G_{max}}=b+\frac{a}{1+e^{\left(\frac{V0.5–V}{k}\right)}},$$

from which the slope factor (k) and half-maximal activation voltage (V_0.5_) were derived. A standard double-pulse protocol was used to generate steady-state inactivation curves for I_Na_. Normalized peak inward currents were plotted as a function of the conditioning potentials and the data fitted to an appropriate Boltzmann function:

$$\frac{I_{Na}}{I_{max}}=b+\frac{a}{1+e^{\left(\frac{V–V0.5}{k}\right)}},$$

from which the slope factor (k) and half-maximal inactivation voltage (V_0.5_) were derived.

### NEP exposure

**Fig 1:**
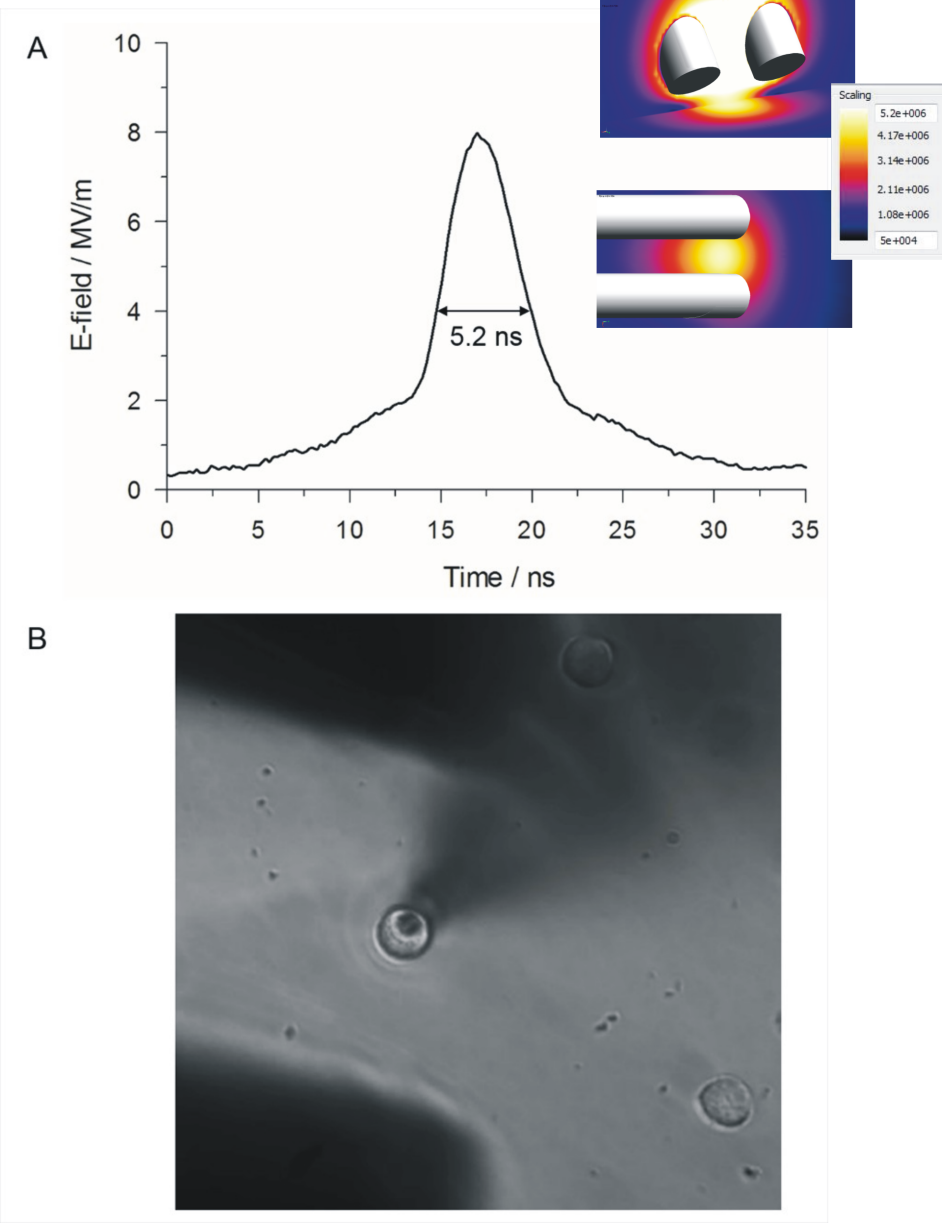
NEP exposure of a chromaffin cell. (A) Representative waveform of a 5 ns, 8 MV/m pulse. The inset shows the simulated E-field distribution between the electrodes and at the level of the patch clamped cell on a color-coded scale as viewed from the side (top) and from above (bottom) the electrodes. (B) Photomicrograph of a patched chromaffin cell located between the electrode tips that are positioned 40 μm above the bottom of the dish.

A nanosecond pulse generator, designed and fabricated by Transient Plasma Systems, Inc. (Torrance, CA), produced pulses that were 5 ns in duration (Fig 1A) that were delivered to a pair of cylindrical, gold-plated tungsten rod electrodes (127 μm diameter) having a gap of 100 μm between the electrode tips. After rupturing the plasma membrane to achieve the whole-cell recording mode, the NEP-delivering electrodes were lowered to a predefined “working” position using the automated targeting function of a motorized MP-225 micromanipulator (Sutter Instruments, Novato, CA). In this “working” position, the patched cell was situated midway between the electrode tips, with electrode tips positioned 40 μm from the bottom of the coverslip (Fig 1B). A single pulse was applied to the cells at E-field amplitudes ranging from 5 to 10 MV/m. The E-field distribution at the location of the target cell was computed using the commercially available Finite-Difference Time-Domain (FDTD) software package SEMCAD X (version 14.8.5, SPEAG, Zurich, Switzerland) and is shown in the inset of Fig 1A. The two simulated images show that the E-field was uniformly distributed at the plane of the cell during the application of a NEP. Whole-cell membrane currents were continuously recorded, except for the interval between 20 ms before and 8 ms after delivery of a pulse, using an automated pulse exposure system controlled by a program written in LabVIEW [22].

### Statistical analysis

For all experiments, results were obtained using cells from different days in culture and different cell preparations and presented as the mean ± standard error (SE). For all NEP experiments, each cell was exposed to a single NEP. With the exception of experiments in which we explored the effects of NEPs on inward and outward currents at a +10 mV test potential, all data originated from independent experiments. In experiments examining inward and outward currents at +10 mV, the data were collected from the same cells at each E-field tested. The data were analyzed with SPSS software using either a paired Student’s t test when the means of two groups were compared, or a One-Way ANOVA test for repeated measures followed by Tukey post hoc multiple range tests in multiple group comparisons. P < 0.05 was considered statistically significant.

### Reagents

Ham’s F-12, dispase II and the antibiotics-antimycotics were obtained from Gibco Laboratories (Grand Island, NY, USA), bovine calf serum was purchased from Gemini Bio-products (West Sacramento, CA, USA), and collagenase B was obtained from Roche Diagnostics (Indianapolis, IN, USA). All other chemicals were reagent grade and purchased from standard commercial sources.

## Results

### Properties of macroscopic ionic currents in bovine chromaffin cells

**Fig 2:**
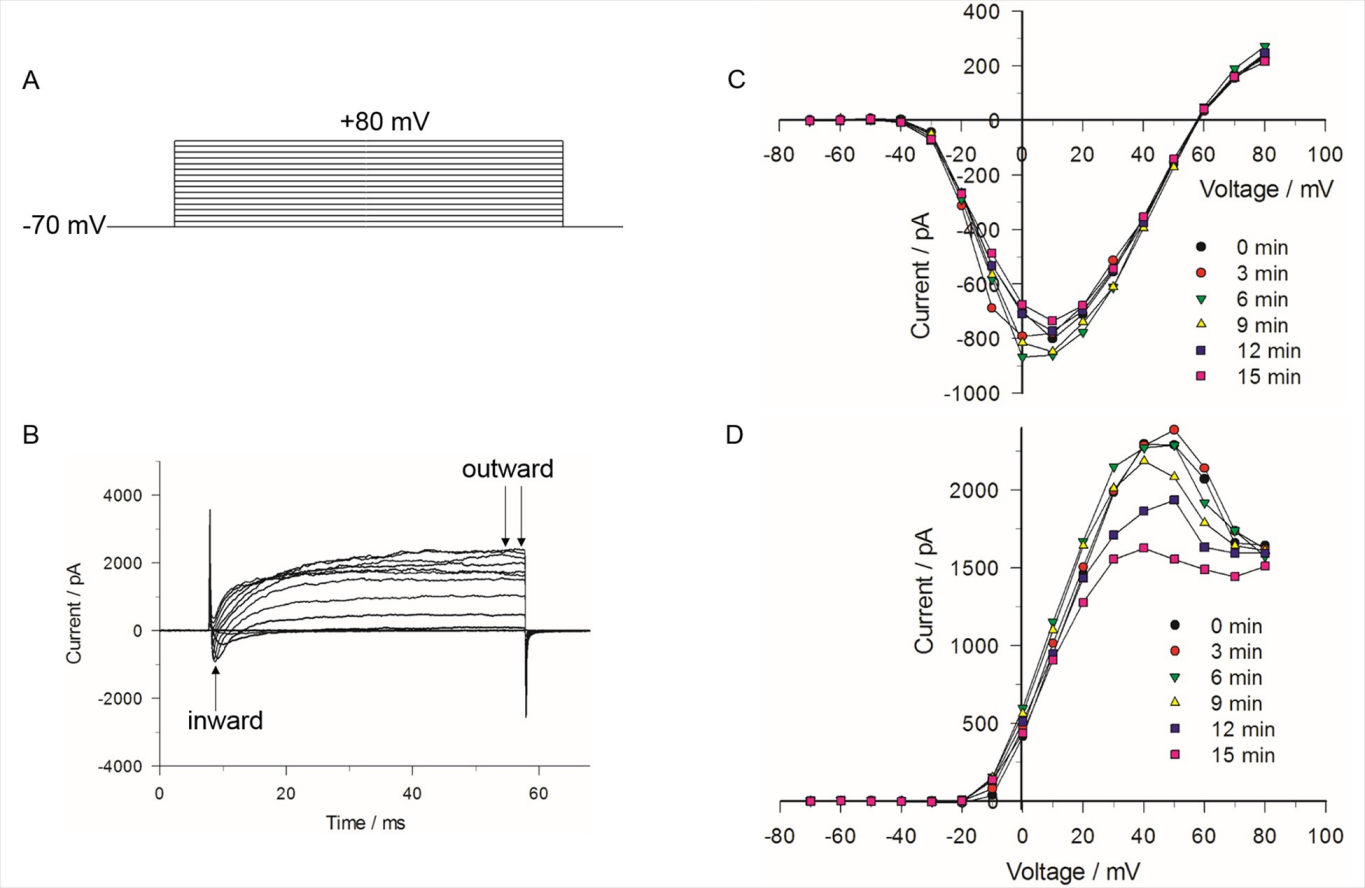
I-V relationships for early peak inward and late outward currents. (A) The voltage step protocol consisted of 50 ms steps ranging from –70 to +80 mV, applied in 10 mV increments every 2 s. (B) Corresponding traces of the early inward and late outward current elicited by the voltage step protocol. (C) I-V relationship for the peak inward current (arrow in (B)) obtained for each of 6 successive voltage step protocols applied to the same cell. The interval between each protocol was 3 min for a total elapsed time of 21 min. (D) Corresponding I-V relationship for the outward current of the same cell shown in (C). Current represents the mean value of the late outward current for the interval between the arrows in (B). The results are representative of 5 cells.

Preliminary experiments were carried out to characterize the general biophysical properties of peak inward and late outward currents recorded in whole-cell voltage clamped chromaffin cells using the voltage step protocol shown in Fig 2A. The pipette and bathing solutions with physiological pH and isotonic salt concentration were set to allow for recording membrane currents under near “physiological conditions”. The traces below the voltage protocol in Fig 2B represent a typical family of membrane currents characterized by an early transient inward current followed by a late outward current. Fig 2C and 2D show a series of I-V relationships for peak inward and late outward currents (measured as shown in Fig 2B), respectively, recorded in the same cell at 3 min intervals. The early transient inward current activated near –30 mV, peaked around +10 mV and reversed at ~ +56 mV. The late outward current activated near –20 mV and displayed a bell-shaped voltage-dependence with a peak observed around +40 mV. While the voltage-dependence and amplitude of the inward current remained stable over the course of 10 min or more (in some cases more than 30 min), the outward current measured between 0 and +70 mV ran down in tens of seconds to a few minutes after seal rupture, which contrasted with outward currents elicited by moderate (e.g. –10 mV) and strong (e.g. +80 mV) depolarizations. These results are consistent with previous studies in these cells [26,27] showing that the late outward current is composed of a mixture of ionic currents comprising: 1) a voltage-dependent Ca^2+^ current (I_Ca_), 2) a Ca^2+^-activated K^+^ current (I_K(Ca)_), as evident from the bell-shaped I-V curve that mirrors that of a typical I_Ca_, and 3) a delayed and sustained rectifier K^+^ current (I_KV_). The outward current at +40 mV is a mixture of a small voltage-dependent Ca^2+^ current, a large conductance Ca^2+^-activated K^+^ current (I_K(Ca)_), itself activated by Ca^2+^ entry through Ca^2+^ channels, and a sustained voltage-dependent delayed rectifier K^+^ current (I_KV_). Since activation of I_K(Ca)_ is triggered by I_Ca_, the rundown of the outward current observed between 0 and +70 mV would be consistent with the well-known rundown of several types of voltage-gated Ca^2+^ channels (e.g. L-type Ca^2+^ channels encoded by Ca_V_1 subunits) [31–33] when recorded in the whole-cell patch clamp configuration [26,34]. The lack of rundown of the outward current at potentials below 0 mV and above +70 mV would be consistent with the stable properties of voltage-dependent delayed rectifier K^+^ channels [27] under these conditions.

**Fig 3:**
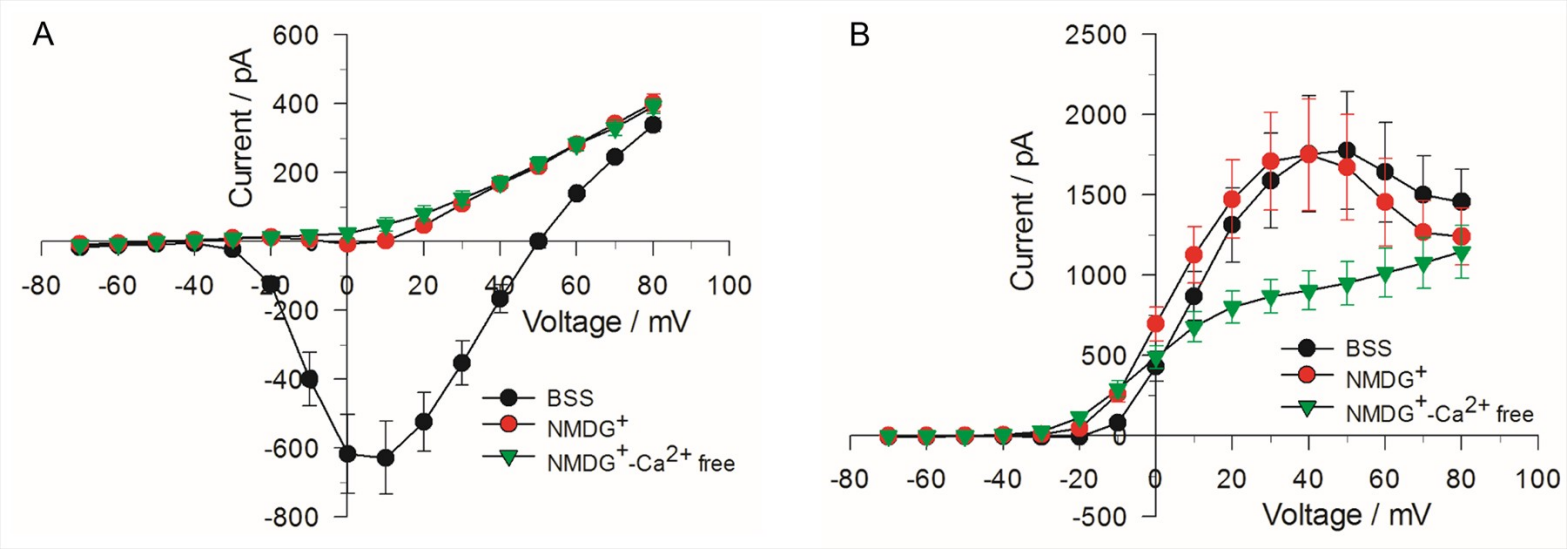
Effect of eliminating Na^+^ and Ca^2+^ on the I-V relationships for inward and outward currents. The voltage step protocol was the same as described in Fig 2 for obtaining inward (A) and outward (B) currents. The I-V relationship was first obtained in the presence of normal BSS, then after the external bath solution was changed to BSS in which extracellular Na^+^ was absent (NMDG^+^) and finally to BSS in which both extracellular Na^+^ and Ca^2+^ were absent (NMDG^+^-Ca^2+^free). The data represent the mean value of 4 cells ± SE.

Ion replacement experiments were performed to define the ionic nature of the inward and outward currents registered in our experiments. As shown in Fig 3A, total replacement of extracellular Na^+^ with the non-permeant ion NMDG^+^ nearly abolished the inward current, indicating it is predominantly carried by Na^+^, with barely detectable and slower I_Ca_ that was converted to net outward current when both Na^+^ and Ca^2+^ were removed from the external solution. As described in a later section, the half-inactivation potential of V_0.5_ (~ –45 mV) determined from the analysis of the steady-state inactivation properties of the inward current is also consistent with this current being predominantly composed of a fast voltage-dependent Na^+^ current (I_Na_) [26,35]. Total replacement of Na^+^ with NMDG^+^ also produced a significant leftward shift (~ –6 mV) of the bell-shaped voltage-dependence of the outward current, an observation consistent with the previously reported negative shift of the steady-state activation curve of Ca^2+^ current produced by substitution of internal NMDG^+^ for Cs^+^ [36]. Removal of both external Ca^2+^ and Na^+^ converted the bell-shaped I-V curve to a sigmoidal curve typical of that recorded for delayed rectifier K^+^ channels (Fig 3B). Taken together these results confirm that the inward current is largely determined by I_Na_ whereas the outward current reflects the properties of a small inward I_Ca_ activating I_K(Ca)_, superimposed with I_KV_ [26,27].

### A single pulse reduces I_Na_

**Fig 4:**
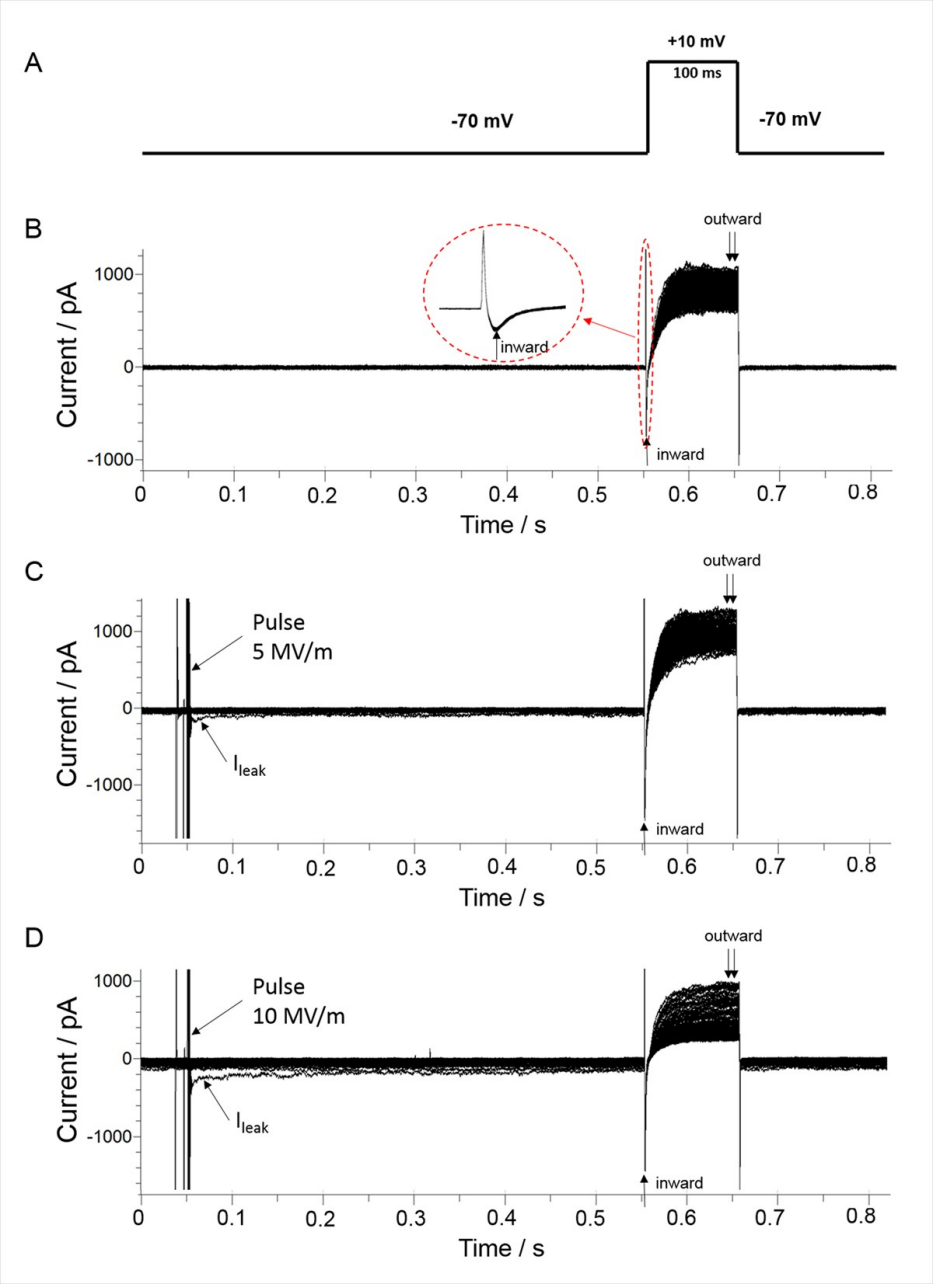
Inward and outward currents evoked by a constant-voltage step protocol. (A) The constant-voltage step protocol consisted of applying a 100 ms voltage step to +10 mV from a holding potential. (B) Control traces of inward and outward current obtained by applying a total of 200 voltage step protocols, with a 3 s interval between each protocol. The inset shows an expanded view of the peak inward current. (C) Representative traces of inward and outward current following exposure of a cell to a single 5 ns pulse applied at an E-field of 5 MV/m, and in (D), at an E-field of 10 MV/m. In both (C) and (D), the pulse was applied between the 20^th^ and 21^st^ voltage step protocols, with an interval of 0.5 s between the time the NEP was delivered and recording of currents. The arrow indicates I_leak_, which was recorded with a delay of 8 ms after the pulse [22].

A constant-voltage step protocol (Fig 4A) was used to monitor the time course of changes on I_Na_ in cells exposed to a 5 ns pulse. The protocol consisted of stepping membrane potential from –70 mV to +10 mV every 3 s for 10 min. The test potential of +10 mV was selected because it nears the peak of the Na^+^ conductance (Fig 2C). We first determined I_Na_ stability in the absence of a NEP (Fig 4B). We next tested the effect of a single NEP on I_Na_ elicited by the same constant-voltage step protocol. Membrane currents were first recorded for 60 s prior to NEP exposure to establish baseline conditions. A single NEP applied at an E-field of either 5 MV/m (Fig 4C), 8 MV/m or 10 MV/m (Fig 4D) was then delivered between the 20^th^ and 21^st^ voltage steps of the 200 voltage step train. Consistent with our previous whole-cell recordings obtained in patch clamped cells [22], the NEP caused a small instantaneous inward leak current (I_leak_) at the holding potential (Fig 4C and 4D) that decayed exponentially in an E-field strength-dependent manner.

**Fig 5:**
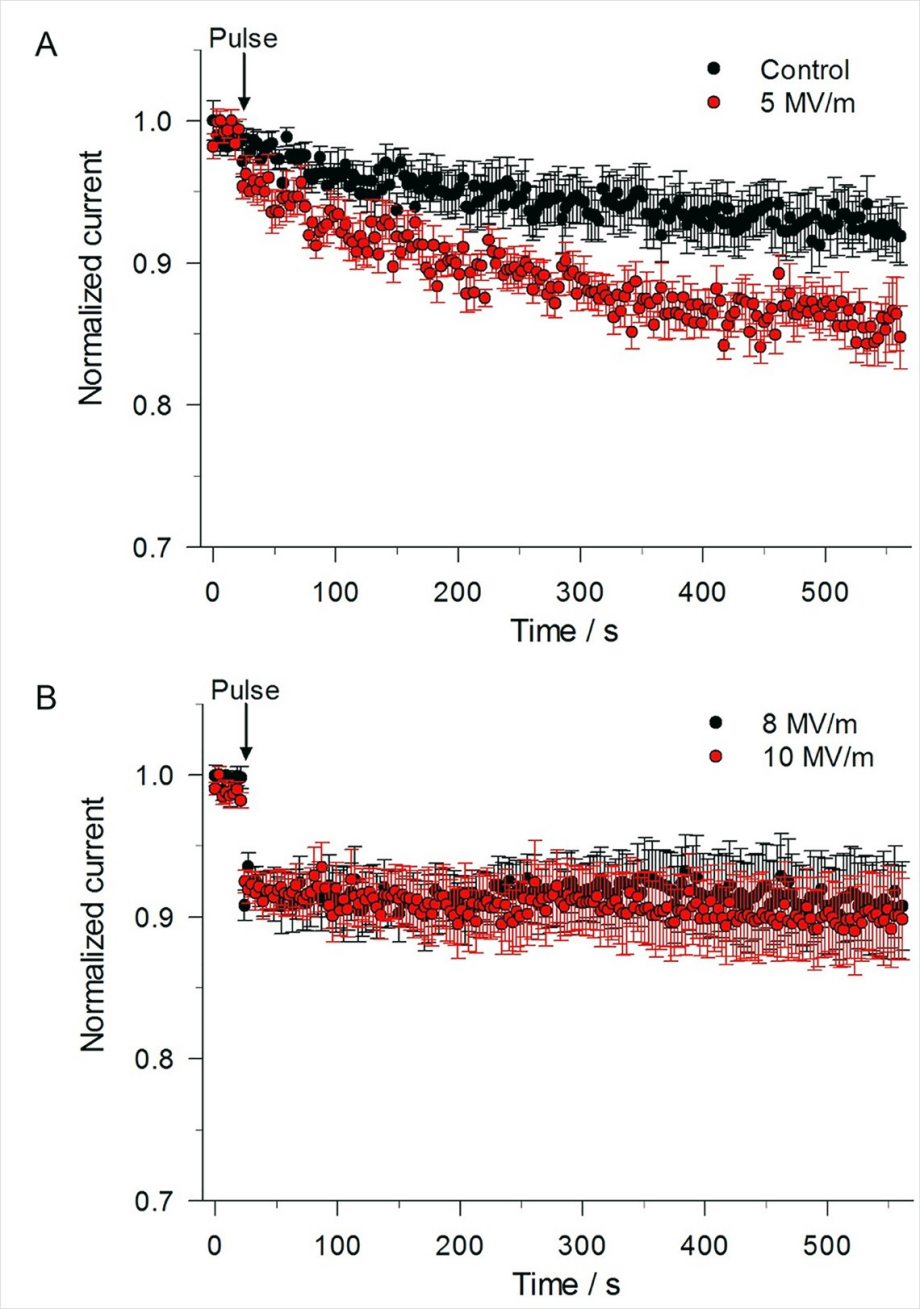
Effect of a single 5 ns pulse at different E-field amplitudes on peak inward current. (A) Time course of the changes in peak inward current for an unexposed cell (control) compared to a cell exposed to a 5 ns, 5 MV/m pulse, obtained by using the constant-voltage step protocol described in Fig 4. Normalized current represents the magnitude of peak inward current normalized to the mean of the peak inward current recorded for the 8 voltage step protocols that immediately preceded the 21^st^ voltage step protocol. Data are expressed as the mean ± SE (control, n = 11; 5 MV/m, n = 9). (B) Time course of the changes in peak inward current for a cell exposed to a 5 ns pulse at E-fields of 8 MV/m and 10 MV/m. Data are expressed as the mean ± SE (8 MV/m, n = 10; 10 MV/m, n = 11).

The time course profile of the changes in I_Na_ resulting from NEP exposure are shown in Fig 5. In the absence of a NEP, the inward current at +10 mV slowly declined over a period of ~10 min to a level that was ~92% of the initial level (Fig 5A). The small decline in I_Na_ in these experiments may be attributable, at least in part, to the small but significant contribution of I_Ca_ to peak inward current as suggested by ion replacement experiments (Fig 3A). A single NEP applied at an E-field of 5 MV/m (Fig 5A) caused a sudden ~4% decrease in peak I_Na_ recorded 0.5 s after the pulse. I_Na_ then declined exponentially to a level that was ~85% of the initial level after 10 min. When the E-field amplitude was increased to 8 or 10 MV/m (Fig 5B), the inhibitory effect on peak I_Na_ recorded 0.5 s after the pulse was twofold greater (~9%) in magnitude in each case. There was no further decline in I_Na_ over the course of 10 min, indicating that the effect of the pulse on I_Na_ was saturated at the higher E-field amplitudes. One-Way ANOVA revealed a significant difference among the groups. I_Na_ recorded from cells exposed to 5 MV/m (P< 0.001), 8 MV/m (P< 0.001) and 10 MV/m (P< 0.001) were all significantly different from the control group. However, there was no significant difference detected between data collected from cells exposed to the three E-field intensities (5 MV/m vs. 8 MV/m, P = 0.069; 5 vs. 10 MV/m, P = 0.068; 8 MV/m vs. 10 MV/m, P = 1.000).

### Is I_leak_ reducing I_Na_ by eliciting a voltage drop across the series resistance?

**Fig 6:**
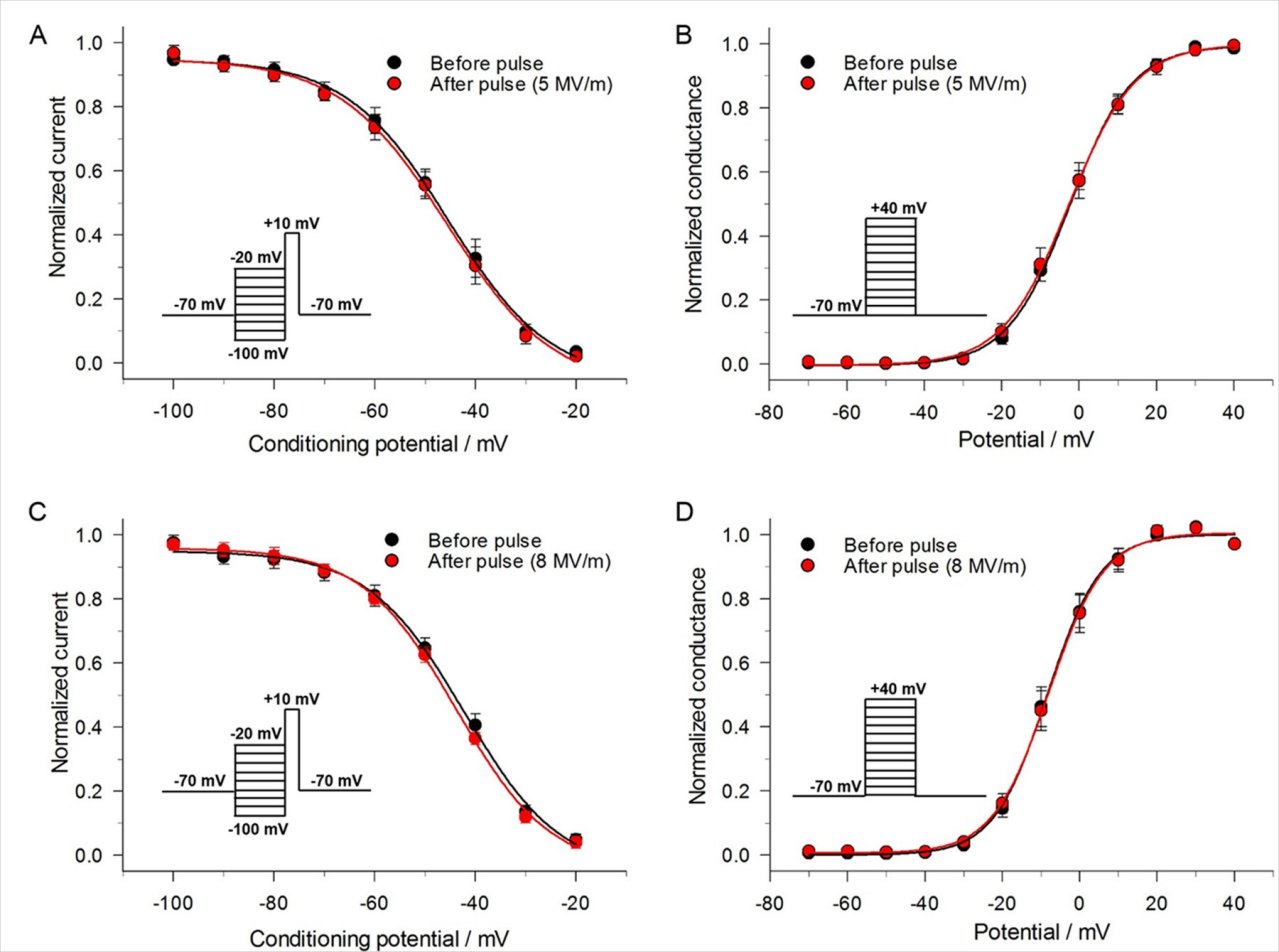
Effect of a single 5 ns pulse on the voltage-dependence of steady-state inactivation and activation of I_Na_. (A) Steady-state inactivation of I_Na_ was determined by holding cells at –70 mV and applying a series of 1 s conditioning potentials ranging from –100 mV to –20 mV in 10 mV increments, with each voltage step followed by a constant 100 ms test pulse to +10 mV to record I_Na_ (inset). A 5 MV/m pulse was delivered and after 1 s the voltage step protocol repeated. I_Na_ in each case was normalized to their respective maximal values and plotted as a function of the conditioning potential. The data were fitted to a Boltzmann function (see Methods) and expressed as the mean ± SE (n = 4). (B) Steady-state activation of Na^+^ at an E-field of 5 MV/m. Steady-state activation of I_Na_ was determined by holding cells at –70 mV and applying 50 ms steps ranging from –70 mV to +40 mV in 10 mV increments every 2 s (inset). A 5 MV/m pulse was delivered and after 1 s the voltage step protocol repeated. Peak Na^+^ current in each case was converted to conductance (see Methods), normalized to their respective maximal values and fitted to a Boltzmann function. Data are expressed as the mean ± SE (n = 6). (C) Same as in (A) for a pulse applied at an E-field of 8 MV/m (n = 6). (D) Same as in (B) for a pulse applied at an E-field of 8 MV/m (n = 5).

When using the whole-cell patch clamp method, the command voltage (V_c_) is distributed across the series resistance (R_s_) and the cell membrane resistance (R_m_), yielding a clamped membrane potential (V_m_) that is actually less than V_c_. The presence of an I_leak_ evoked by the NEP at the holding potential could cause a greater difference between V_m_ and V_c_ by eliciting a voltage drop across R_s_. Such an effect could cause current error measurements of I_Na_. To determine the extent to which I_leak_ affected I_Na_, we first calculated the voltage error (V_Err_) that would be attributed to a voltage drop across R_s_ produced by the I_leak_ measured just prior to recording I_Na_. The calculations were based on the equation V_Err_ = I_leak_ * R_s_ and the results are shown in Table 1 for an NEP applied at 5, 8 or 10 MV/m. At each E-field amplitude, I_leak_ exerted only a small effect on R_s_ wherein V_Err_ was 0.6 ± 0.2 mV, 0.7 ± 0.1 mV and 1.0 ± 0.1 mV for 5, 8 and 10 MV/m, respectively. We next determined the impact of these voltage errors on I_Na_ by quantifying their effect on the steady-state inactivation curve of I_Na_ shown in Fig 6A. For an NEP of 5 MV/m, the 0.6 mV depolarization caused by I_leak_ decreased I_Na_ by 0.5%, which is much less than the ~4% inhibition of I_Na_ evoked by the pulse (Fig 5A). For an NEP of 8 MV/m, the depolarization caused by I_leak_ decreased I_Na_ also by 0.5%, considerably less than the ~9% inhibition of I_Na_ evoked by the pulse (Fig 5B). These results demonstrate that I_leak_ had only a small effect on V_Err_ and hence recordings of I_Na_, which in turns means that the inhibitory effect of the NEP on I_Na_ was not the result of a voltage drop across R_s_.

**Table 1:** Decrease in I_Na_ caused by I_leak_ versus the NEP.

E-Field (MV/m)	Rs^a^ (MΩ)	Rs^b^ (MΩ)	I_leak_ (pA)	V_Err_ (mV)	ΔI_Na_^c^ (%)	ΔI_Na_^d^ (%)
**5 (n = 9)**	12.7 ± 1.5	13.9 ± 1.2	43 ± 17	0.6 ± 0.2	0.5 ± 0.2	4.3 ± 0.5
**8 (n = 9)**	10.3 ± 2.2	10.9 ± 1.6	66 ± 5	0.7 ± 0.1	0.5 ± 0.1	9.2 ± 0.9
**10 (n = 8)**	12.9 ± 1.2	13.9 ± 1.4	71 ± 5	1.0 ± 0.1	─	9.2 ± 0.3

^a^ Before and

^b^after the NEP; for 5 MV/m, P = 0.122; for 8 MV/m, P = 0.465; for 10 MV/m, P = 0.065.

^c^ Decrease caused by V_Err_ and calculated from the I_**Na**_ inactivation curve.

^d^ Decrease immediately after delivery of the NEP.

### A single pulse has no effect on the voltage-dependence of inactivation and activation of I_Na_

The inhibitory effect of a 5 ns pulse on I_Na_ could also be caused by a negative shift of the steady-state inactivation curve, a positive shift of the activation curve, or both. In order to test for possible changes in steady-state inactivation, cells were held over a range of potentials for 1 s and then depolarized to a test pulse (+10 mV) to evaluate the availability of Na^+^ channels. Fig 6A and 6C show the effect of a NEP at two E-field strengths (5 and 8 MV/m) on the voltage-dependence of inactivation. For a NEP of 5 MV/m, half-maximal inactivation voltages V_0.5_ were –45.4 ± 0.9 mV (k = –10.1 ± 0.8) and -45.6 ± 1.3 mV (k = –10.6 ± 1.1) before and after the pulse (P = 0.07, n = 4), respectively. For an NEP of 8 MV/m, V_0.5_ were –42.2 ± 1.3 mV (k = –9.5 ± 1.0) and –43.8 ± 0.8 mV (k = –9.3 ± 0.6) before and after the pulse (P = 0.11, n = 6), respectively. Thus, regardless of E-field amplitude, there was no significant effect of the NEP on the steady-state inactivation of I_Na_.

Steady-state activation curves for I_Na_ were constructed by measuring peak Na^+^ current during the different test depolarization potentials (similar to Fig 2A) and then converting the value to Na^+^ conductance (G_Na_). Our results indicate that a single 5 ns pulse at 5 or 8 mV/m produced no significant effect on the voltage-dependence of activation of I_Na_ (Fig 6B and 6D). Half-maximal activation voltages V_0.5_ were –2.7 ± 0.3 mV (k = 8.5 ± 0.3) and –2.5 ± 1.3 mV (k = –10.6 ± 1.1) before and after a 5 MV/m NEP (P = 0.85, n = 6). The E_rev_ (data not shown) were –55.7 ± 1.3 mV and –55.3 ± 1.8 mV before and after the pulse (P = 0.57, n = 6), respectively. When the NEP was applied at 8 MV/m, V_0.5_ were –8.4 ± 0.5 mV (k = 6.9 ± 0.4) and –8.1 ± 0.5 mV (k = 7.1 ± 0.4) before and after the pulse (P = 0.44, n = 5), respectively, and the E_rev_ (data not shown) were –54.4 ± 0.9 mV and –53.9 ± 1.0 mV before and after the pulse, respectively (P = 0.70, n = 5).

### A single pulse decreases maximal Na^+^ conductance

**Fig 7:**
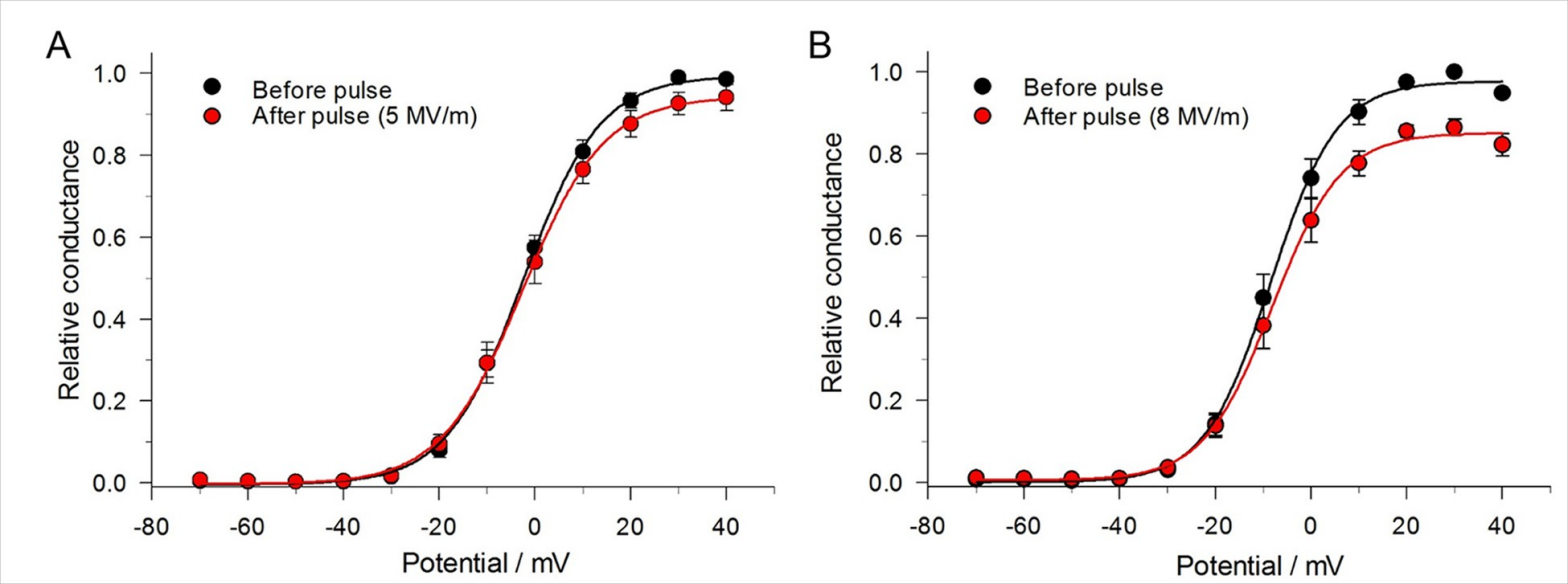
Effect of a single 5 ns pulse at different E-field amplitudes on maximal Na^+^ conductance. The protocol (inset) was the same as that for generating steady-state activation of I_Na_. Peak I_Na_ was converted to conductance as already described and relative conductance obtained by normalizing the conductance both before and after the pulse to the maximum conductance before the pulse. The data were fitted to a Boltzmann function (see Methods) and expressed as the mean ± SE. (A) Pulse applied at an E-field of 5 MV/m (n = 6) and (B) at 8 MV/m (n = 5).

Another possibility that could account for the NEP-induced decrease of peak I_Na_ is a decrease in maximal conductance of the Na^+^ channel. To assess this possibility, we determined the difference in absolute Na^+^ conductance before and after a single pulse. For this determination, relative Na^+^ channel conductance was obtained by normalizing the conductance of the channel after the pulse to the conductance of the channel measured before the pulse. As shown in Fig 7, a single 5 ns pulse reduced relative channel conductance. At +40 mV, the decrease in maximal Na^+^ conductance was 5% and 15% at an E-field of 5 MV/m and 8 MV/m, respectively, indicating that the magnitude of the effect was dependent on the E-field amplitude.

### A single pulse causes variable inhibitory effects on outward current

**Fig 8:**
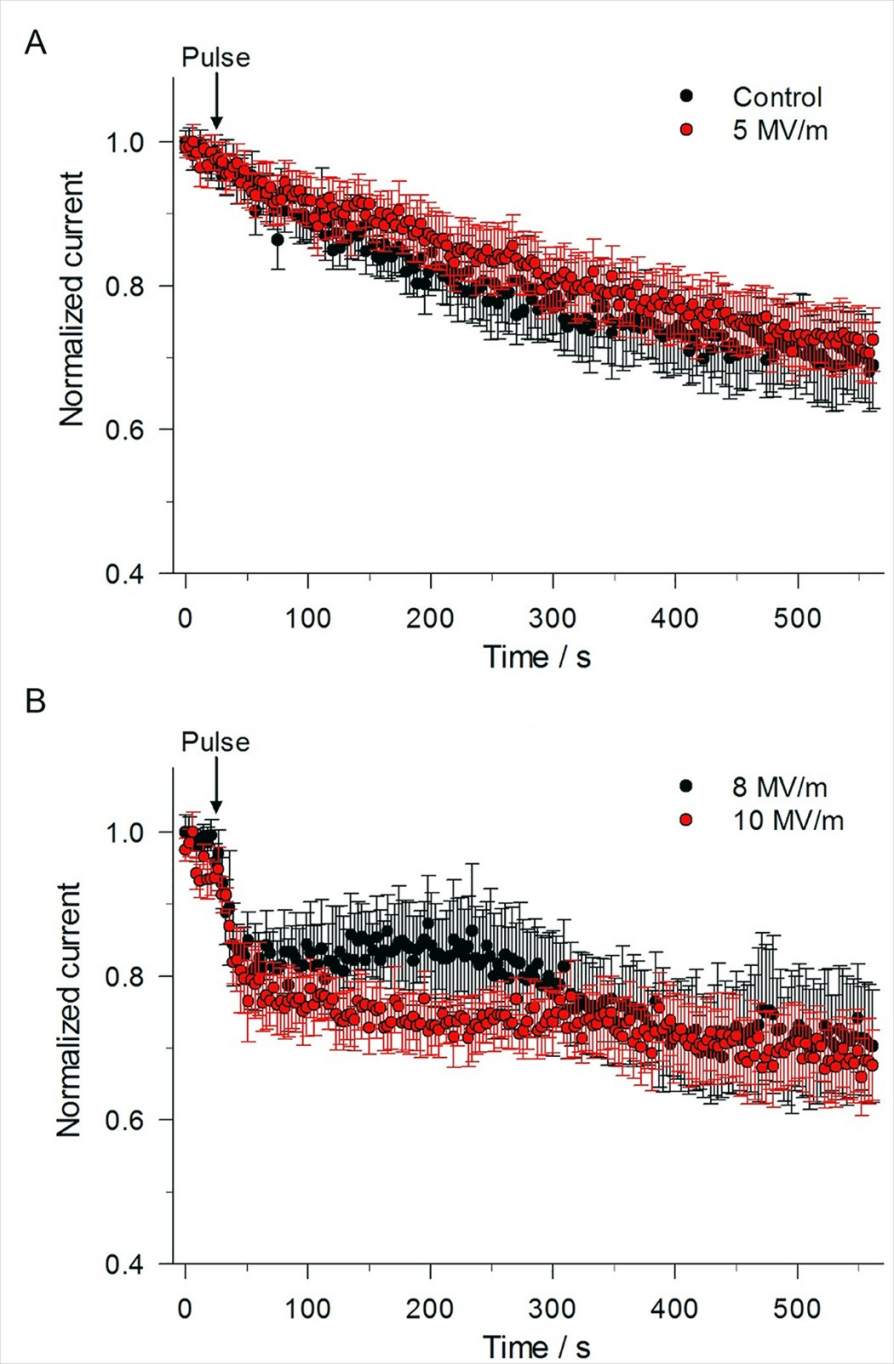
Effect of single 5 ns pulse at different E-field amplitudes on late outward current. (A) Time course of the changes in mean outward current for an unexposed cell (control) compared to a cell exposed to a 5 ns, 5 MV/m pulse, obtained by using the constant-voltage step protocol described in Fig 4A in which the voltage was stepped from –70 mV to +10 mV for 100 ms. Normalized current represents the mean magnitude of the late outward current (see Fig 4B) normalized to the mean of the late outward current recorded for the 8 voltage step protocols that immediately preceded the 21^st^ voltage step protocol. Data are expressed as the mean ± SE (Control, n = 11; 5 MV/m, n = 9). (B) Time course of the changes in mean outward current for a cell exposed to a 5 ns pulse at E-fields of 8 MV/m and 10 MV/m (8 MV/m, n = 3/10; 10 MV/m, n = 6/11). In both (A) and (B), the results were obtained from the same cells as those shown in Fig 5 for peak inward current.

The same constant-voltage step protocol that was used to monitor the time course of changes on I_Na_ (Fig 4A) in cells exposed to a 5 ns pulse was used to monitor NEP effects on the late outward current. Control experiments revealed that the late outward current measured at +10 mV displayed prominent rundown, as shown in Fig 8A. This greater rate of decline in outward current is consistent with the rundown described for the I-V relationships shown in Fig 2D, a behavior that is consistent with I_Ca_ rundown and the progressive attenuation of the Ca^2+^ trigger for activation of outward I_K(Ca)_ [27]. Fig 8A shows that the application of a single pulse at an E-field of 5 MV/m had no effect on the late outward current. When the E-field amplitude was increased from 5 to 8 MV/m, the outward current of 3 out of 10 cells underwent a progressive 20–25% decline over the course of the first 20 s after the pulse. The outward current then stabilized at this reduced level over the course of 10 min (Fig 8B). There was no effect of the pulse on the other 7 cells. When the E-field amplitude was increased from 8 to 10 MV/m, a greater proportion of cells (6 of 11 cells) responded to the NEP (Fig 8B) but the magnitude and time course of inhibition was similar to that observed with 8 MV/m.

**Fig 9:**
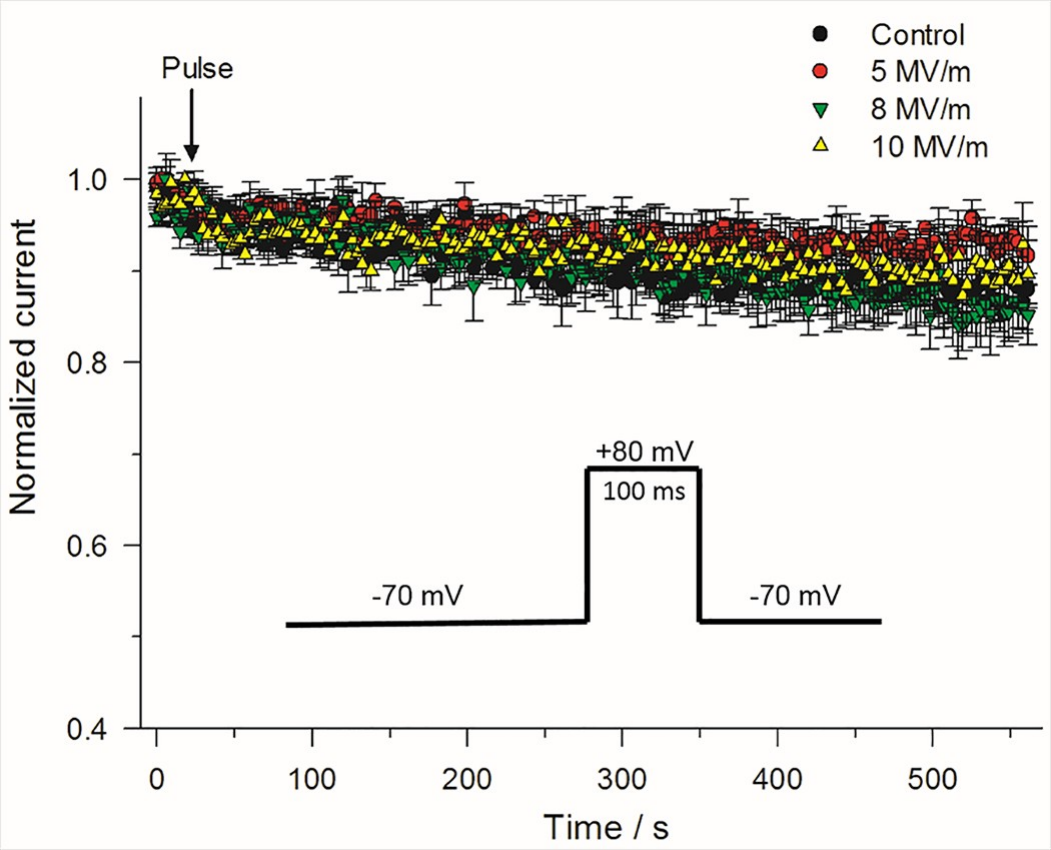
Effect of a single 5 ns pulse at different E-field amplitudes on late outward current. Time course of the changes in mean outward current for an unexposed cell (control) compared to a cell exposed to a 5 ns pulse applied at E-fields of 5, 8 and 10 MV/m. The constant-voltage step protocol consisted of stepping the voltage from –70 mV to +80 mV for 100 ms (inset). Normalized current represents the same as that described in Fig 8. Results are expressed as the mean ± SE (Control, n = 4; 5 MV/m, n = 5; 8 MV/m, n = 5; 10 MV/m, n = 5).

As discussed before, at the test potential of +10 mV, the outward current is a mixture of a voltage-dependent Ca^2+^ current, a Ca^2+^-activated K^+^ current (I_K(Ca)_) and a sustained voltage-dependent delayed rectifier K current (I_KV_). Thus, it is difficult to distinguish the effect of the NEP on these superimposed currents. To separate NEP effects on outward currents, a revised constant-voltage step protocol was developed. Voltage steps to +80 mV instead of +10 mV were applied every 3 s while maintaining holding potential. At the test potential of +80 mV, the outward current would be primarily composed of the more stable voltage-dependent delayed rectifier K^+^ current with little contamination from I_Ca_ and I_K(Ca)_ due to the greatly reduced driving force for Ca^2+^ and thus the much smaller impact of I_Ca_ on I_K(Ca)_. The control experiment in Fig 9 clearly shows that the outward current at +80 mV was much more stable than that recorded at +10 mV (Fig 8), declining only by ~10% over 10 min. When a single pulse at an E-field of 5 MV/m, 8 MV/m or 10 MV/m was applied to the cells, there was no significant effect of the pulse on the outward current at +80 mV (Fig 9), indicating that the decrease in outward current observed at the test potential of +10 mV was primarily concentrated on I_Ca_ and I_K(Ca)_ as I_KV_ was not affected.

## Discussion

The results of this study have shown that a single high-intensity 5 ns electric pulse produces differential E-field-dependent inhibitory effects on voltage-gated cation channels in bovine chromaffin cells. Fast transient inward Na^+^ current was the most sensitive ionic current displaying inhibition in response to a NEP at E-fields ≥ 5 MV/m. This effect was not due to a shift in the voltage-dependence of steady-state activation or inactivation but was associated with a reduction in maximal Na^+^ conductance. In contrast, a single NEP inhibited outward K^+^ current at higher field intensities (≥ 8 MV/m) but the effect was voltage-dependent, with inhibition detected in a fraction of cells at +10 mV, and no inhibition observed at +80 mV even at an E-field of 10 MV/m. The potential cellular targets, molecular mechanisms and therapeutic implications of these findings are discussed.

### Experimental strategy and limitations

The experimental approach used in this study exploited conditions designed to simulate near physiological Na^+^ and K^+^ gradients and took advantage of a novel NEP delivery system allowing for the near continuous recording of whole-cell macroscopic currents with a gap of only 28 ms when exposing a chromaffin cell to a 5 ns pulse. This system significantly reduces the delay time from pulse exposure to resumption of cell membrane recording compared to other studies (delay times ranging from 10 seconds to 2 minutes) [5,37], which minimizes missing important whole-cell monitoring information immediately after pulse delivery. Voltage clamp protocols were devised to examine the effects of a single NEP on several ionic currents in the same chromaffin cell, which eliminated potential disparities in channel sensitivity related to differences in experimental conditions and batches of cells. This approach was further supported by the comprehensive body of literature on the biophysical properties of ion channels in this well studied cell model.

We first established that the net inward current elicited by depolarizing steps from a holding potential was mainly produced by voltage-gated Na^+^ channels as the current was nearly abolished by replacing external Na^+^ with the non-permeant NMDG^+^. Removing external Ca^2+^ in the presence of NMDG^+^ only led to a small further reduction in inward current (discernable by a small increase in net outward current) that was less than 8% of peak current. The gating properties of net inward current were also consistent with those of tetrodotoxin-sensitive Na^+^ channels in chromaffin cells [35], and of Na_V_1.7, the pore-forming Na^+^ channel subunit thought to be the main voltage-gated Na^+^ channel expressed in these cells [38].

Repetitive steps to +10 mV were used to monitor the time-dependent effects of a NEP on membrane currents. This voltage was initially selected because it elicited near maximal Na^+^ conductance, while also activating voltage-dependent Ca^2+^ and K^+^ currents. The current measured at the end of the voltage steps was likely predominantly composed of a Ca^2+^-activated K^+^ current (I_K(Ca)_) and a voltage-dependent delayed rectifier K^+^ current (I_KV_) [27], to which a small partially inactivated Ca^2+^ current (I_Ca_) was superimposed. The bell-shaped voltage-dependence of late outward current is consistent with K_Ca_ channels being triggered by Ca^2+^ influx through “neighboring” Ca^2+^ channels since: 1) the shape of the I-V relationship mirrored that predicted for I_Ca_ in these cells and was apparent in cells dialyzed with a high concentration of the Ca^2+^ chelator EGTA [27]; 2) the bell-shaped voltage-dependence of the outward current was converted to a sigmoidal relationship following external Ca^2+^ removal [27,39], with or without external Na^+^; and 3) similar to many types of high-threshold, voltage-dependent Ca^2+^ channels, the late outward current exhibited pronounced rundown in the range of +10 to +70 mV. This has also been reported by Marty and Neher [27] in the same preparation. In contrast, the current at +80 mV was much more stable and thus primarily reflected the activity of I_KV_ since the driving force for Ca^2+^ would be very small and would thus produce very weak stimulation of I_K(Ca)_. This argument is also supported by the observation that removal of external Ca^2+^ had no effect on this current at +80 mV in the absence of Na^+^ and justified our rationale of examining the effects of NEP at this voltage in separate experiments.

### Effects of NEP on voltage-gated Na^+^ channels

Our data provided evidence for an E-field-dependent inhibition of I_Na_ following a single 5 ns pulse. Although smaller in magnitude, the effects were nevertheless similar to those of Pakhomov et al. [24,25] who reported that much longer nanosecond pulses (300 and 600 ns) were able to modulate voltage-gated Na^+^ and Ca^2+^ channels in GH3, NG108 and even chromaffin cells. Our data showed that I_Na_ decreased instantaneously by ~4% and then declined exponentially following a single 5 ns, 5 MV/m pulse while higher E-fields produced instantaneous inhibitory effects that were significantly larger with no further decline (~9% at E-fields of 8 and 10 MV/m) over the course of 10 min. We first considered the possibility that the inhibition might be due to an alteration in the voltage-dependence of activation and/or inactivation. Our results clearly showed that NEPs up to 8 MV/m produced no significant effect on either property, suggesting that the ultrashort electric pulse did not interfere with Na^+^ channel gating. We did find that the NEP reduced maximal Na^+^ chord conductance and potential mechanisms to explain this observation are discussed below.

High intensity NEPs of less than 1 μs in duration (from 5 up 600 ns) were shown to evoke a transient “leak” conductance that is hypothesized to be formed by ion-permeable nanoelectropores [22,24,25]. The sudden appearance of a nanopore or “leak” conductance (I_leak_) just prior to recording I_Na_ after delivery of the NEP could have potentially lowered V_m_ sufficiently to depolarize the command holding potential set to –70 mV due to a voltage drop across the uncompensated R_s_ and thus reduce Na^+^ channel availability despite a lack of change in the voltage-dependence of inactivation. However, the potential impact of this voltage error (V_Err_) on peak I_Na_ based on the steady-state inactivation curve revealed that the depolarization would have decreased I_Na_ by only 0.5% both at 5 MV/m and at 8 MV/m, which is significantly less than that observed. These results suggest that V_Err_ associated with activation of I_leak_ due to a voltage drop across R_s_ was too small to explain the much larger inhibition of I_Na_ evoked by NEPs at any E-field magnitude.

Recent experiments from our group confirmed that the plasma membrane of chromaffin cells becomes permeable to Na^+^ following activation of I_leak_ evoked by a single 5 ns pulse [22]. Thus, intracellular accumulation of Na^+^ could potentially account for the inhibition of I_Na_ by reducing the electrochemical gradient for Na^+^. However, this seems unlikely in view of the fact that the reversal potential of the net inward current was also not altered by NEPs. These results suggest that the reduction of I_Na_ was not associated with a change in ion selectivity. This conclusion is in agreement with the results of Nesin and Pakhomov [25] who concluded that the much larger Na^+^ influx through I_leak_ evoked by longer NEPs could not explain the reduction of I_Na_.

The pipette solution used in our experiments contained a high concentration of the Ca^2+^ chelator EGTA, which would argue against but cannot exclude the possibility that the NEP-induced decrease of I_Na_ was caused by an intracellular Ca^2+^-dependent process since EGTA is known to be a slow Ca^2+^ buffer (e.g., the buffering capacity of this chelator was insufficient to prevent activation of I_K(Ca)_ triggered by I_Ca_). However, consistent with the idea that intracellular Ca^2+^ was not involved was the observation that inhibition of I_Na_ by 300 ns NEPs was unaffected by cell dialysis with 20 mM BAPTA, a much faster chelator [25].

In this study, we confirmed that a single NEP reduced maximal I_Na_ conductance, which can be defined by *G*_*max*_ = N * g_Na_* P_*Omax*_, where N is the total number of Na^+^ channels in the membrane, g_Na_ is the unitary conductance of Na^+^ channels and P_Omax_ is the maximum open probability of Na^+^ channels. The 5 ns pulse could reduce maximal conductance by altering one or more of these parameters. Single-channel experiments will be required to determine which of these factors is influenced by the NEP. There are at least three possible mechanisms for explaining the inhibitory effects of an NEP on Na^+^ channels: 1) the NEP affects the Na^+^ channel protein directly; 2) the NEP affects the structure of the phospholipid environment (e.g. disruption of lipid rafts and caveolae, the distribution of cholesterol, etc.), which indirectly alters their activity; or 3) both. Direct effects of longer duration electric pulses (4 ms) on voltage-gated channels were previously reported by Chen et al. [40,41]. They showed that a single 4 ms transmembrane potential shock of –400 mV or –450 mV decreased Na^+^ and K^+^ channel conductance and proposed that membrane proteins were somehow damaged by an unknown denaturation process. The lipid bilayer of the cell plasma membrane is another primary target that can be affected by externally applied electric fields [1,42,43]. Previous studies have shown that membrane disturbances caused by NEPs initiate complex intracellular lipid signaling pathways [44]. Changes in the biochemical and biophysical properties could alter channel activity and membrane excitability in response to activation of receptors [44]. Phosphoinositides, especially phosphatidylinositol (4,5)-bisphosphate or PIP_2_, serve as signature motifs for different cellular membranes and often are involved in the modulation of multiple types of ion channels [45,46]. It has been demonstrated that 600 ns electric pulses can initiate hydrolysis or depletion of PIP_2_ in the plasma membrane [44], which could be responsible for the NEP-induced inhibition of voltage-gated channels [25]. Therefore, disruption of the phospholipid bilayer by an NEP could be a possible step leading to subsequent inhibition of voltage-gated channels. More experiments will be required to test this hypothesis.

### Effects of NEP on voltage-gated K^+^ channels

Single NEPs also inhibited outward K^+^ currents elicited at +10 mV but this effect was only detectable in a fraction of cells at higher E-fields (8 and 10 MV/m) compared to that observed on I_Na_ (E-field of 5 MV/m). In contrast, the outward K^+^ current evoked at +80 mV was not influenced by NEPs up to 10 MV/m. For the reasons stated above, the K^+^ current elicited at this voltage is primarily composed of I_KV_. Nesin et al. [24] reported that a single 600 ns NEP inhibited I_Ca_ in GH3 cells but this effect required a higher E-field than that produced by a single 300 ns pulse on I_Na_ recorded in NG108 cells, suggesting that voltage-gated Ca^2+^ channels, as our study would suggest for Ca^2+^-activated K^+^ currents measured at +10 mV, are less sensitive to NEPs. Clearly more experiments will have to be carried out to determine whether 5 ns NEPs selectively inhibited I_Ca_, I_K(Ca)_ or both.

### Potential implications

Potassium, calcium, and sodium channels play critical roles in the development of major diseases, such as hyperkalemia, epilepsy, congenital myotonia and serious neurological, retinal, cardiac, and muscular disorders [47–50]. On this basis, the inhibition of voltage-gated channels has potential medical applications. NEPs may lower excitability in nerve cells and block nerve conduction, mimicking the activity of local anesthetics and nerve blocking agents [51–53]. Recently, the role of voltage-sensitive ion channels (potassium, calcium, and sodium channels) has been linked to the progression of cancer and these channels are becoming the targets of significant drug developmental efforts to modulate voltage-sensitive ion channel activity in order to prevent or combat malignant disease [47]. The inhibition of I_Na_ and I_Ca_ has also been shown to have important roles in cell adhesion, invasiveness, angiogenesis and chronic pain relief [54–57]. Thus, the inhibition of voltage-gated channels with NEPs, especially in light of possible differences in sensitivity to E-fields, has potential implications in cancer treatment. In addition, the development of NEPs to modulate ion channels directly or indirectly in excitable and non-excitable cells is likely to become a promising therapeutic avenue with great potential for medical benefits in the near future.
